# Assessment of long-term quality of life in patients with anal carcinomas treated by radiotherapy with or without chemotherapy

**DOI:** 10.1038/sj.bjc.6690567

**Published:** 1999-07

**Authors:** A S Allal, M A G Sprangers, F Laurencet, M A Reymond, J M Kurtz

**Affiliations:** Division of Radiation Oncology and Department of Surgery, University Hospital of Geneva, 1211 Geneva 14, Switzerland; Department of Medical Psychology, Academic Medical Center, 1105 Amsterdam, The Netherlands

**Keywords:** anal carcinoma, quality of life, radiotherapy, chemotherapy

## Abstract

This study was conducted to assess long-term Quality of Life (QOL) in patients treated by radiotherapy with or without chemotherapy for anal carcinomas. Patients with a maximum age of 80 years, and who were alive at least 3 years following completion of treatment with a functioning anal sphincter and without active disease, were selected for this study. Of 52 such patients identified, 41 (79%) were evaluable. There were 35 females and six males with a median age of 71 years (55–80). The median follow-up interval was 116 months (range 37–218). QOL was assessed using two self-rating questionnaires developed by the European Organization for Research and Treatment of Cancer: one for cancer-specific QOL (EORTC QLQ-C30) and one for site-specific QOL (EORTC QLQ-CR38). For the function scales a higher score represents a higher level of functioning (100 being the best score), whereas for the symptom scales a higher score indicates a higher level of symptomatology/problems (0 being the best score). For the QLQ-C30, the functional scale scores ranged from 71 (global quality of life) to 85 (role function) and the symptom scale scores from 6 (nausea-vomiting) to 28 (diarrhoea). For the QLQ-CR38 module the functional scale scores ranged from 13 (sexual functioning) to 74 (body image) and for the symptom scale scores from 5 (weight loss) to 66 (sexual dysfunction in males). None of the functional and symptom scale scores seemed to be better in patients with longer follow-up. In patients treated with sphincter conservation for anal carcinomas, long-term QOL as measured by the EORTC QLQ-C30 and QLQ-CR38 appears to be acceptable, with the exception of diarrhoea and perhaps sexual function. Moreover, the subset of patients who presented with severe complications and/or anal dysfunction showed poorer scores in most scales. © 1999 Cancer Research Campaign


*British Journal of Cancer* (1999) **80**(10), 1588–1594
© 1999 Cancer Research Campaign
Article no. bjoc.1999.0567

### Assessment of long-term quality of life in patients with anal carcinomas treated by radiotherapy with or without chemotherapy

**AS Allal[1], MAG Sprangers[3], F Laurencet[1], MA Reymond[2] and JM Kurtz[1]**

[1]Division of Radiation Oncology and [2]Department of Surgery, University Hospital of Geneva, 1211 Geneva 14, Switzerland; [3]Department of Medical Psychology, Academic Medical Center, 1105 Amsterdam, The Netherlands

**Summary** This study was conducted to assess long-term Quality of Life (QOL) in patients treated by radiotherapy with or without chemotherapy for anal carcinomas. Patients with a maximum age of 80 years, and who were alive at least 3 years following completion of treatment with a functioning anal sphincter and without active disease, were selected for this study. Of 52 such patients identified, 41 (79%) were evaluable. There were 35 females and six males with a median age of 71 years (55–80). The median follow-up interval was 116 months (range 37–218). QOL was assessed using two self-rating questionnaires developed by the European Organization for Research and Treatment of Cancer: one for cancer-specific QOL (EORTC QLQ-C30) and one for site-specific QOL (EORTC QLQ-CR38). For the function scales a higher score represents a higher level of functioning (100 being the best score), whereas for the symptom scales a higher score indicates a higher level of symptomatology/problems (0 being the best score). For the QLQ-C30, the functional scale scores ranged from 71 (global quality of life) to 85 (role function) and the symptom scale scores from 6 (nausea-vomiting) to 28 (diarrhoea). For the QLQ-CR38 module the functional scale scores ranged from 13 (sexual functioning) to 74 (body image) and for the symptom scale scores from 5 (weight loss) to 66 (sexual dysfunction in males). None of the functional and symptom scale scores seemed to be better in patients with longer follow-up. In patients treated with sphincter conservation for anal carcinomas, long-term QOL as measured by the EORTC QLQ-C30 and QLQ-CR38 appears to be acceptable, with the exception of diarrhoea and perhaps sexual function. Moreover, the subset of patients who presented with severe complications and/or anal dysfunction showed poorer scores in most scales.

**Keywords:** anal carcinoma; quality of life; radiotherapy; chemotherapy

The curability of the majority of anal carcinomas using radiotherapy, especially when administered in combination with chemotherapy, has been convincingly demonstrated (Papillon et al, 1974; Anonymous, 1996; Bartelink et al, 1997). Abdominoperineal resection (APR) has consequently fallen into disfavour in the initial management of this disease, particularly since the anatomical advantage offered by sphincter-conserving approaches is assumed to be associated with definite quality of life (QOL) advantages. Beside the preservation of the anatomical integrity of normal structures, QOL of patients surviving anal cancer may be influenced by additional factors, including treatment-related side-effects and the physiological function of the preserved organs. Indeed, conservative approaches based on radiotherapy, with or without chemotherapy, can be associated with chronic complications that may significantly impair QOL. Moreover, while major late complications that require APR or diverting colostomy occur in less than 15% of treated patients (Papillon et al, 1989; Touboul et al, 1994; Allal et al, 1997), pelvic irradiation may potentially be associated with functional symptoms related to anorectal dysfunction in a more significant proportion of cases (Sedgwick et al, 1994; Yeoh et al, 1996). QOL of patients with certain pelvic malignancies has been assessed after various treatment approaches (Gelber et al, 1996; Anderson and Lutgendorf, 1997). However, there are thus far no published reports concerning QOL outcome in patients treated with sphincter conservation for anal carcinoma. The aim of this single-institution cross-sectional study was to evaluate long-term QOL in patients treated with such approaches and to try to identify factors that might negatively affect QOL parameters.

*Received 2 November 1998*
*Revised 10 February 1999*
*Accepted 22 February 1999*

*Correspondence to:* AS Allal

### PATIENTS AND METHODS

### Patients characteristics

The study population was drawn from among 165 patients with anal carcinoma who received sphincter-conserving treatment using radiotherapy, with or without chemotherapy, between January 1976 and December 1994 at the Geneva University Hospital. All patients were considered for QOL assessment who were 80 years old or less at the time of the study, and who were alive without disease activity at least 3 years after completion of treatment with a functioning anal sphincter. The maximum age limit was chosen to avoid a significant impact of the comorbidities on QoL, or on the validity of its assessment. Fifty-two patients satisfied the inclusion criteria. Forty-nine patients were contacted by telephone to solicit their participation, and three who had no telephone number were contacted by mail. Forty-six patients gave

**Table 1** Patient characteristics

| Parameters | Number of patients |
|---|---|
| Median initial age, years (range) | 60 (42–75) |
| Median actual age, years (range) | 71 (55–80) |
| Gender: male/female | 6/35 |
| Tumour location | |
| Canal | 30 |
| Margin | 3 |
| Canal ± margin ± rectum | 8 |
| Histology | |
| Keratinizing squamous | 26 |
| Basaloid and transitional | 15 |
| TNM classification (UICC, 1987)[a] | |
| T1/T2 | 6/18 |
| T3/T4 | 16/1 |
| N0 | 30 |
| N1–3[b] | 11 |

[a]Anal canal classification. [b]Six patients with inguinal nodes.

**Table 2** Characteristics of radiotherapy

| | | Median dose/ fraction/days |
|---|---|---|
| Fields and doses (1st course) | | |
| AP/PA pelvic fields (+ laterals) | 32 (+2) | 40 Gy/20/31 |
| Perineal ± sacral fields | 7 | 30 Gy/10/20 |
| Boost technique (2nd course) | | |
| (A) EBRT | 10 | 20 Gy/10/12 |
| Perineal field | 5 | |
| AP/PA ± lateral fields | 3 | |
| Other techniques | 2 | |
| (B) Interstitial brachytherapy | 31 | |
| Median dose – median dose rate | 20 Gy – [illegible] Gy/h | |
| Median interval between courses | 39 days | |
| Median total treatment duration | 72 days | |

their approval to participate in the study, one refused, two were judged ineligible because of serious co-morbidities and the three patients contacted by mail did not respond. Among the 46 patients who received the two questionnaires, five refused to complete them for different reasons (unclear, two; number of questions, two; questions related to sexual aspects, one), leaving 41 (79%) patients evaluable for the present analysis. Patient characteristics are displayed in Table 1. The median follow-up time was 116 months (range 37–218).

### Treatment

Details of treatment techniques have been described in a previous report (Allal et al, 1993). Eleven patients received radiotherapy alone and 30 concomitant radiation and chemotherapy. In all cases radiotherapy was delivered in two sequences. The first sequence was designed to treat involved sites and the potential microscopic involved areas and consisted of external beam radiotherapy (EBRT) with 60 Co when Papillon's technique was used (perineal field ± sacral field), or with photons of 6 MV or more when antero-posterior opposed pelvic fields were used. The second sequence 'boost' directed to the initial involved sites consisted of brachytherapy in 31 patients and EBRT in ten. Radiotherapy treatment details are displayed in Table 2.

Chemotherapy consisted in all cases of 5-fluorouracil (600–800 mg $m^{-2}$ $day^{-1}$ × 4) and Mitomycin-C (10 mg $m^{-2}$), except in two instances where Mitomycin-C was replaced by leucovorin or cisplatin. Generally, chemotherapy started on day 1 and consisted of 1 cycle in 25 patients, 2 cycles in four patients and 3 cycles in one patient. The doses of the different agents were adjusted according to the age and general condition of the patients.

Before radiotherapy, six patients had an excisional biopsy and 3 an inguinal adenectomy. One patient who presented with a local recurrence after radiotherapy was salvaged by a limited local surgery. The remainder of the patients had not undergone any surgical treatment for anal cancer.

### QOL assessment

The assessment of QOL was performed by using two questionnaires developed by the QOL Study Group of the European Organization for Research and Treatment of Cancer: a validated questionnaire assessing cancer-specific QOL (EORTC QLQ-C30) (Aaronson et al, 1993) and one assessing site-specific (colorectal) QOL (EORTC QLQ-CR38), which is in the process of validation.

### *EORTC QLQ-C30*

This is a patient self-rating questionnaire that comprises six multi-item function scales measuring physical, role, social, emotional and cognitive functions, and overall QOL. Separate symptom scales are included to assess pain, fatigue and emesis, and five single items to measure gastrointestinal symptoms, dyspnoea, appetite loss and sleep disturbances. A final item evaluates the perceived economic consequences of the disease.

### *EORTC QLQ-CR38*

This module is a patient self-rating questionnaire that comprises 38 questions, of which 19 are completed by all patients and the remaining by subset of patients (males or females; patients with or without a stoma). The general structure comprises four multi-item/single-function scales, seven multi-item symptom scales and one single symptom item. The functional scales assess body image, sexual functioning, sexual enjoyment and future perspective. The symptom scales assess radiotherapy side effects on micturition, chemotherapy side-effects, gastrointestinal general symptoms, defecation problems, stoma-related problems and sexual dysfunction in males or females. The single symptom item assesses weight loss. This module has been validated in The Netherlands (Sprangers MAG, Velde te A, Aaronson NK, on behalf of the European Organisation for Research and Treatment of Cancer Study Group on Quality of Life. The construction and testing of the EORTC Colorectal Cancer Specific Quality-of-Life questionnaire Module QLQ-CR38, manuscript under review) and is currently being used in a wide range of cross-cultural studies.

Two supplementary questions were added to the questionnaire to assess the degree of satisfaction with anorectal function and patients' current preferences regarding treatment modalities (conservative vs APR), taking into consideration the functional outcome. The first question used an analogue scale from 1 (totally dissatisfied) to 10 (totally satisfied). For the second question, patients had the choice between three answers: 1 = I still prefer the sphincter-conserving procedure; 2 = sometimes I think an APR

© 1999 Cancer Research Campaign

**Table 3** EORTC QLQ-C30 mean scale and single items scores for the Geneva University Hospital (GUH) and the Danish Central Population Register (DCPR) series

| | GUH series, *n* = 41 [Standard Deviation] | Women population-based sample, DCPR series *n* = 608 |
|---|---|---|
| Functional scales | | |
| Physical function | 79.5 [22] | 86 (80)[a] |
| Role function | 85 [21] | 88 (85) |
| Emotional function | 77 [25] | 77 (79) |
| Cognitive function | 76 [23] | 85 (82) |
| Social function | 82 [28] | 91 (91) |
| Global quality of life | 71 [21] | 72 (70) |
| Symptom scales | | |
| Fatigue | 27 [22] | 25 (29) |
| Pain | 15 [21] | 21 (24) |
| Nausea and vomiting | 6 [15] | 4 (4) |
| Single items | | |
| Dyspnoea | 13 [22] | 9.5 (11) |
| Sleep disturbance | 23.5 [29] | 23 (28) |
| Appetite loss | 10 [19] | 6 (7) |
| Diarrhoea | 28 [36] | 7 (7) |
| Constipation | 15 [21] | 8 (9) |
| Financial impact | 15 [28] | 7 (8) |

[a]Value in the brackets are the scores for women aged 51–75 years.

might have been preferable; and 3 = I definitely think an APR would have been preferable. In addition, by discussion with the patient, anal sphincter function was evaluated according to the Memorial Sloan-Kettering-Cancer Center anal function criteria (MSK-AF) (Minsky et al, 1992). The score ‘excellent = 1’ corresponds to 1–2 bowel movements per day and no soilage; ‘good = 2’ corresponds to 3–4 bowel movements per day and/or mild soilage; ‘fair = 3’ corresponds to episodic >4 bowel movements per day and/or moderate soilage and finally ‘poor = 4’ corresponds to incontinence. Late complications were classified according to the RTOG grading system (Perez and Brady, 1992).

### Statistical methods

All scores of the QLQ-C30 and QLQ-CR38 are linearly transformed such that all scales range from 0 to 100. The higher scale score represents a higher level of functioning for the six (QLQ-C30) and four (QLQ-CR38) multi-item/single-function scales and a higher level of symptomatology/problems for the symptom/single-item scales. Missing values were calculated such that if at least half the items from the scale had been completed, it was assumed that the missing items would have values equal to the average of those present items.

The Mann–Whitney *U*-test was used to assess for significant differences in score medians between subgroups. A difference with a *P*-value ≤0.05 was considered as significant. The choice of a non-parametric test was based on the score distributions that were restricted to the upper middle part of the functioning scales and to the lower or middle parts of the symptom scales. All factors studied, except gender, were selected to define groups of at least ten patients. We hypothesized that at least some scores of the various scales would vary between subgroups of patients according to some clinical parameters commonly believed to affect QOL such as age, gender, late complications or organ dysfunction and time since treatment. However, for T-stage and the two therapeutic factors (addition of chemotherapy, and type of boost), the study was rather exploratory and no a priori hypotheses were formulated. The Fisher’s exact test was used to assess the relationship between the different factors.

**Table 4** EORTC QLQ-C30: functional and symptom scale score means (s.d.) according to clinical and therapeutic factors

| Factors | Nb Patients | Physical function | Role function | Emotional function | Social function | Overall quality of life | Fatigue | Pain |
|---|---|---|---|---|---|---|---|---|
| Age (current) | | | | | | | | |
| ≤71 | 21 | 85 (19) | 86 (23) | 72 (28) | 82 (31) | 69 (26) | 27 (22) | 14 (24) |
| >71 | 20 | 73 (23) | 84 (19) | 82 (19) | 81 (25) | 73 (15) | 28 (22) | 17 (19) |
| Gender | | | | | | | | |
| Female | 35 | 80 (22) | 86 (20) | 78 (25) | 82 (26) | 73 (21) | 27 (22) | 15 (22) |
| Male | 6 | 73 (20) | 77 (25) | 69 (17) | 83 (40) | 61 (18) | 31 (20) | 17 (21) |
| T stage | | | | | | | | |
| T1–2 | 24 | 77 (24) | 84 (23) | 81 (21) | 82 (30) | 74 (24) | 27 (24) | 13 (21) |
| T3–4 | 17 | 82 (18) | 85 (18) | 71 (28) | 82 (24) | 67 816) | 28 (17) | 18 (22) |
| Treatment stratégy | | | | | | | | |
| RT alone | 11 | 72 (24) | 80 (18) | 72 (32) | 86 (21) | 73 (22) | 25 (20) | 23 (25) |
| RT + CT | 30 | 82 (21) | 86 (22) | 79 (21) | 80 (30) | 70 (21) | 28 (22) | 13 (20) |
| RT plan | | | | | | | | |
| EBRT + brachytherapy | 31 | 80 (21) | 84 (22) | 73 (25) | 77 (30) | 68 (22) | 29 (23) | 18 (23) |
| EBRT alone | 10 | 76 (26) | 88 (15) | 88 (17) | 96 (7) | 80 (13) | 21 (16) | 7 (11) |
| Late complications | | | | | | | | |
| Grade 0–1 | 11 | 83 (21) | 94 (15) | 88 (15) | 91 (17) | 85 (15)* | 20 (18) | 11 (20) |
| Grade 2–4 | 30 | 78 (22) | 81 (22) | 73 (26) | 79 (30) | 66 (21) | 30 (22) | 17 (22) |
| MSK anal function score | | | | | | | | |
| Score 1 | 21 | 80 (23) | 92 (13) | 75 (28) | 87 (21) | 76 (22)* | 25 (17) | 17 (23) |
| Score 2–4 | 20 | 79 (22) | 77 (25) | 79 (20) | 76 (33) | 66 (19) | 29 (25) | 14 (20) |
| Follow-up (months) | | | | | | | | |
| ≤116 | 21 | 78 (25) | 82 (24) | 84 (17) | 83 (31) | 72 (18) | 26 (25) | 11 (17) |
| >116 | 20 | 81 (19) | 87 (17) | 69 (29) | 80 (24) | 70 (24) | 29 (18) | 20 (25) |

[a]*P* ≤ 0.05; s.d.: standard deviation; RT: Radiotherapy; CT: Chemotherapy; EBRT: External Beam RT; GI: Gastro-Intestinal; MSK: Memorial Sloan Kettering.

 © 1999 Cancer Research Campaign

### RESULTS

### EORTC QLQ-C30 scores

The general results for all patients are given in Table 3. The mean scores of the scales that would potentially be affected by the selected clinical and therapeutic parameters are displayed in Table 4. The results are detailed according to the significance level of the differences in the scores between subgroups or the clinical relevance of certain findings. The physical function scale scores did not differ significantly in the subgroups, although older patients tended to report lower scores ($P = 0.08$). For the role function scale, while non-significant, the severity of late complications and poor MSK anal function appeared to have a negative effect ($P = 0.08$ for both). This score did not differ with the length of follow-up. For the emotional and the social function scales, no significant differences were noted between the different subgroups. However, the overall quality of life score was significantly affected by the severity of late complications ($P = 0.005$) and the anal function score ($P = 0.04$). This score did not differ with the current age categories or with the length of follow-up.

No significant differences were noted between subgroups concerning the fatigue and pain symptom scales, particularly according to the length of follow-up.

### EORTC QLQ-CR38 scores

The general results for all patients are given in Table 5. The mean values of the main scales scores are displayed in Table 6 according to selected clinical and therapeutic factors. In the latter Table only scales that would potentially be affected by the selected parameters and that had a satisfactory response rate were selected. Body image function score was significantly lower only in patients with advanced T-stage ($P = 0.003$). For the future perspective function scale, no significant differences were noted between subgroups, while lower scores were reported in patients with higher grade of late complications ($P = 0.1$). The sexual functioning score was significantly lower only in advanced age subgroup ($P = 0.01$). None of the functional scale scores seemed to be influenced by the length of follow-up.

Micturition dysfunction symptom scores were significantly higher in patients treated with a brachytherapy boost ($P = 0.02$) and in patients with long follow-up ($P = 0.02$). No significant differences in the scores of general gastrointestinal symptoms

**Table 5** EORTC QLQ-CR38 mean functional scale and symptom scores

| Scales | Nb. patients | Scores (s.d.) |
|---|---|---|
| Functional scales | | |
| Body image | 41 | 74 (29) |
| Future perspective | 41 | 62 (30) |
| Sexual functioning | 40 | 13 (20) |
| Sexual enjoyment | 8 | 66 (25) |
| Symptom scales | | |
| RT side-effects on micturition | 41 | 28 (18) |
| Chemotherapy side-effects | 30 | 16 (20) |
| General gastrointestinal | 41 | 21 (17) |
| Defecation problems | 41 | 18 (14) |
| Sexual dysfunction of males | 6 | 66 (31) |
| Sexual dysfunction of females | 8 | 18 (14) |
| Weight loss | 41 | 5 (14) |

RT: radiotherapy; s.d.: standard deviation.

**Table 6** EORTC QLQ-CR38: functional and symptom scale score means (s.d.) according to clinical and therapeutic factors

| Factors | Nb. patients | Body image | Future perspective | Sexual functioning[b] | Micturition dysfunction | General GI symptoms | Defecation problems |
|---|---|---|---|---|---|---|---|
| Age (current) | | | | | | | |
| ≤71 | 21 | 67 (32) | 60 (31) | 22 (23)[a] | 24 (16) | 21 (14) | 21 (15) |
| >71 | 20 | 80 (25) | 63 (30) | 3 (9) | 32 (20) | 20 (20) | 15 (11) |
| Gender | | | | | | | |
| Female | 35 | 74 (29) | 62 (30) | 13 (20) | 28 (20) | 21 (18) | 17 (12) |
| Male | 6 | 70 (34) | 61 (33) | 16 (21) | 29 (9) | 16 (14) | 23 (22) |
| T stage | | | | | | | |
| T1–2 | 24 | 85 (25)[a] | 68 (30) | 14 (20) | 28 (15) | 20 (17) | 17 (15) |
| T3–4 | 17 | 58 (28) | 53 (29) | 13 (20) | 27 (23) | 22 (18) | 19 (12) |
| Treatment stratégy | | | | | | | |
| RT alone | 11 | 74 (27) | 69 (28) | 10 (17) | 25 (17) | 21 (17) | 15 (11) |
| RT + CT | 30 | 73 (30) | 59 (31) | 14 (21) | 29 (19) | 20 (17) | 19 (14) |
| RT plan | | | | | | | |
| EBRT + brachytherapy | 31 | 75 (28) | 58 (30) | 13 (19) | 31 (19)[a] | 22 (19) | 21 (13)[a] |
| EBRT alone | 10 | 69 (32) | 73 (30) | 13 (22) | 17 (13) | 16 (10) | 10 (11) |
| Late complications | | | | | | | |
| Grade 0–1 | 11 | 79 (31) | 76 (21) | 21 (23) | 24 (14) | 14 (14) | 11 (9)[a] |
| Grade 2–4 | 30 | 72 (29) | 57 (32) | 10 (18) | 29 (20) | 23 (18) | 21 (14) |
| MSK anal function score | | | | | | | |
| Score 1 | 21 | 79 (26) | 68 (23) | 16 (21) | 28 (16) | 19 (14) | 12 (10)[a] |
| Score 2–4 | 20 | 68 (32) | 53 (35) | 11 (19) | 28 (21) | 22 (20) | 24 (14) |
| Follow-up (months) | | | | | | | |
| ≤116 | 21 | 78 (29) | 68 (30) | 13 (22) | 21 (17)[a] | 19 (15) | 19 (17) |
| >116 | 20 | 69 (29) | 55 (29) | 13 (18) | 35 (18) | 22 (20) | 18 (10) |

[a]$P \leq 0.05$; SD: Standard Deviation; RT: Radiotherapy; CT: Chemotherapy; EBRT: External Beam RT; GI: Gastro-Intestinal; MSK: Memorial Sloan Kettering; [b] 40 patients analysed

© 1999 Cancer Research Campaign 

were noted between subgroups. Finally, a significant higher defecation problems score was reported in patients treated with brachytherapy for the boost ($P = 0.03$).

Seventy-one per cent of patients indicated a high degree of satisfaction with their present ano-rectal function (score 7–10), 24% a moderate satisfaction (score 4–6) and 5% a low satisfaction (score 1–3). Regarding treatment preference, despite suboptimal function in some cases, 38 patients (93%) preferred their present status with anal sphincter preserved, while 3 (7%) had at least thought of the possibility that an APR might have been a better choice.

### DISCUSSION

Although the potentially negative impact of APR on QOL has been well studied in patients with rectal cancer (Williams and Johnston, 1983; Sprangers et al, 1995), little is known about QOL parameters in long-term survivors of anal carcinomas following non-surgical sphincter-conserving treatment, despite the wide acceptance of such approaches (Papillon, 1974; Nigro et al, 1974; Anonymous, 1996). Recently, we reported that in this setting, radiotherapy, with or without chemotherapy, may be associated with an actuarial rate of serious late complications as high as 20% at 8 years (Allal et al, 1997). Taking these results into consideration, we undertook a study designed to allow formal assessment of QOL in all patients less than 81 years of age apparently cured at least 3 years post-treatment with an intact anal sphincter. We succeeded in evaluating 79% of potentially eligible patients treated in our institution, using current QOL methodology based on cancer and site-specific questionnaires developed by the EORTC QOL Study Group. The study population is small, and the cross-sectional design precluded an assessment of the effect of treatment on QOL in the individual patient, or of possible changes in QOL as a function of time. Moreover, the patients were treated over a long time period, and the treatments used were somewhat heterogeneous, both regarding radiotherapy techniques and chemotherapy administration. Nonetheless, this study represents a first step in documenting long-term QOL in conservatively-treated anal cancer patients, and may provide insight regarding clinical or treatment factors that negatively influenced QOL parameters.

In the absence of pre-treatment baseline parameters, QOL scores are frequently difficult to interpret. In this regard it may be useful to compare the results obtained in study patients to those determined in a general population. Taking into account that 85% of the patients in our series were female, we compared our patients' scores from the EORTC QLQ-C30 questionnaire with those reported by Klee et al (1997) in 608 Danish women who served as a population-based sample for this questionnaire (Table 3). Interestingly, while cognitive and social function subscales were slightly lower in our patients, probably reflecting their more advanced age, the other functional scales were similar, including global QOL. The only symptom score that was found clearly to be higher in the study patients was diarrhoea, with an apparent threefold increase, reflecting the known association of pelvic irradiation with potentially chronic small intestinal dysfunction (Yeoh et al, 1993). In contrast, the finding of a lower pain symptom score in the study patients was unexpected. However, this score was considered inappropriately high in the Danish series, a finding attributed by the authors to a high prevalence of certain active diseases in the population studied.

Despite the small number of study patients, and the potential problem of multiple testing (possible significant differences due to a type 1 error), we tried to identify subgroups of patients (according to selected factors) reporting lower functional or higher symptom scores. Among the clinical and therapeutic parameters studied, the RTOG late complication grade and the MSK anal function score were the factors that most significantly affect certain QOL scores. Thus, as anticipated from our previous study on late complications (Allal et al, 1997), patients presenting with grade 2–4 complications or MSK-AF scores of 2–4 had significantly lower scores for the overall QOL scale, and these two factors also tended to negatively affect the role function subscale. Moreover, the severity of late complications was associated with a trend to have lower scores for emotional function (irritability and depression) and a higher fatigue symptom score. Considering the chronic aspects of late complications, particularly the irreversibility of anal dysfunction, it is plausible that these factors impact negatively on patients' daily activities and their overall sense of well-being. Although any impact of the length of follow-up must be interpreted with caution, since the scores were determined in different patients receiving non-identical treatments, it is noteworthy that patients with long follow-up did not generally exhibit different QOL profiles from those of patients treated more recently. Nonetheless, one might speculate that the trend toward lower emotional function scores in patients with long follow-up might be a consequence of living for a longer time with chronic complications. Regarding the possible influence of age on general QOL parameters, older patients had similar profiles to those of younger patients, with the exception of a lower physical function score ($P = 0.08$).

No significant impact of treatment variables on general QOL parameters could be demonstrated in the current study. This is not surprising, given the small sample size and the multiple potential interactions between patient-related factors, length of follow-up, radiotherapy technique and chemotherapy administration. A multivariate analysis in a considerably larger patient population would be required to reliably evaluate potential effects of treatment-related variables on QOL. With these reservations in mind, it should be mentioned that patients treated with radiotherapy alone, as well as those patients having had a brachytherapy boost, tended to report a higher pain symptom, and that patients having had brachytherapy showed a trend toward lower scores for the emotional and social function scales. Although we have not found a brachytherapy boost in itself to cause more serious late complications, one can speculate that adjustments of radiotherapy parameters in patients receiving chemotherapy might account for fewer symptomatic sequelae in long-term survivors. In fact, when radiotherapy was used alone, external beam treatment was given with higher dose per fraction (mean 2.43 Gy vs 1.96, for total doses of 36.4 Gy and 39 Gy respectively), and a higher brachytherapy dose was applied (mean 22.5 Gy vs 18 Gy), compared with patients having been treated with concomitant chemotherapy.

Since the site-specific EORTC QLQ-CR38 questionnaire has only recently been validated in The Netherlands for colorectal cancer patients, no meaningful comparisons with other data sets could be provided. The current results (Table 5) have thus been interpreted according to the magnitude of variations from the best theoretical scores, namely 100 for the function scales, and 0 for the symptom scales. Moreover, for some scales we tried to identify factors that seemed to affect the scores (Table 6).

For the body image function scale, the mean score of 74 may be judged as satisfactory, considering the potentially negative impact of alterations in the ano-genital area on body image in both

 © 1999 Cancer Research Campaign females and males (Williams and Johnston, 1983). Patients with stage T3–4 tumours had significantly lower scores, perhaps reflecting a greater tissue volume affected by disease involvement or treatment-related changes. Indeed, while not significant, patients with T3–4 tumours tended to have more severe complications and/or anal dysfunction (data not shown). On the other hand, younger patients (≤ 71 years) tended to have lower scores, perhaps reflecting more preoccupation with their body image than older patients. Combining these two factors, younger patients with T3–4 tumours had a markedly lower body image score (50) compared with older patients with T1–2 tumours (93).

No significant differences were found between subgroups in the future perspective scale score. However, patients with severe complications and/or anal dysfunction had a non-significant trend to have lower scores, and this score seemed to decrease with the length of follow-up. Patients presenting with MSK-AF 2–4 and longer follow-up (>116 months) had a lower score (45) compared with the score (81) of patients with MSK-AF 1 and shorter follow-up. This may reflect the negative effect of persistent complications, particularly chronic anal dysfunction, on the future perspective score.

The sexual functioning score was dramatically low (13). Only 14 patients (35%) reported some sexual activity. Moreover, the extent of this activity varied greatly among patients and never reached the maximum level of functioning in any individual patient. As expected older patients had a significantly lower score compared with younger patients. Also a lower score was observed in patients with severe late complications. Older patients (>71 years) with grade 2–4 complications had a score of 1 compared with the score of 30 observed in younger patients with grade 0–1 complications. Because genital organs are in close proximity to the high-dose treatment volume, the high degree of sexual dysfunction in the present series is in keeping with the results observed in women with gynaecological cancers (Andersen et al, 1989) and men with prostate cancers (Crook et al, 1996), in whom loss of sexual desire and/or orgasm, dyspareunia and loss of potency are frequent. Sexual enjoyment function was reported by only eight women, with the moderate score of 66, consistent with the rather low sexual dysfunction symptom score (18) reported by the women in this study. This is in contrast with the score reported by men (66), reflecting a high degree of sexual dysfunction. The latter score can be considered as surprising, since the nervi erigentis and the pudendal nerve are generally not included in the high dose volume, particularly when brachytherapy is used. Moreover, in the absence of a population-based reference group, it is difficult to determine to which extent the degree of sexual dysfunction is due to treatment in these relatively aged patients.

The overall score for micturition symptom scale was quite high (28) and was significantly higher in patients treated with a brachytherapy boost and in patients with longer follow-up. However, there was a significant relation between these two factors, in that all patients with >116 months' follow-up had received brachytherapy. Since urinary tract complications may become progressively symptomatic over long follow-up (Kapp et al, 1997), it is unclear to what extent brachytherapy in itself truly influences the micturition symptom score.

Considering that all patients received external beam pelvic irradiation, the overall score of 21 for general gastrointestinal symptoms seems acceptable. None of the factors studied significantly influenced this score. Moreover, the score of 18 for defecation problems can be considered as satisfactory, considering the tumour site involved and the rather elderly population studied. As expected RTOG complication grade and MSK-AF score were significantly reflected in the defecation problem scale results (these three parameters may explore the same symptoms). The only treatment factor that significantly affected this score was the use of a brachytherapy boost. While this may represent a real effect, only ten patients were treated with EBRT boosts and their follow-up was shorter. Finally, the weight loss symptom score was very low (5), implying that weight loss is very unlikely to represent a main problem in successfully treated anal cancer patients.

In conclusion, to our knowledge the present study represents the first report on long-term cancer and site-specific QOL in patients treated conservatively by radiotherapy with or without chemotherapy for anal carcinomas. The overall results obtained by using the EORTC QLQ-C30 questionnaire were similar to those of a population-based sample, except for diarrhoea that was observed more frequently in treated anal cancer patients. On the other hand, a clearly negative impact of late complications and/or anal dysfunction on cancer-specific QOL was demonstrated, hence emphasizing the importance of future research aiming at reducing such side-effects. On the basis of the results obtained with the site-specific module (EORTC QLQ-CR38), we conclude that the different function scale scores appear acceptable, with the exception of the low sexual functioning score. In the symptom scale scores gastrointestinal, defecation and micturition dysfunction seemed acceptable, while the sexual dysfunction score was surprisingly quite high, particularly in men. In this regard, while the severity of late complications seems to have a negative impact on some symptom scores, the impact of treatment-related factors merits further exploration, particularly the technical aspects of radiotherapy. For both questionnaires, none of the function and symptom scale scores seem to be improved in patients with longer follow-up. Finally, it is noteworthy that, despite suboptimal anal function in nearly 50% of patients, 71% of patients expressed a high satisfaction with their present anorectal function and only 7% even considered the possibility that APR might have been a preferable approach.

### REFERENCES

Aaronson NK, Ahmedzai S, Bergman B, Bullinger M, Cull A, Duez NJ, Filiberti A, Flechtner H, Fleishman SB, de Haes JC, Kaasa S, Klee M, Osoba D, Rozari D, Rofe PB, Schraub S, Sneeuw K, Sullivan M and Takeda F (1993) The European Organization for Research and Treatment of Cancer QLQ-C30: a quality-of-life instrument for use in international clinical trials in oncology. *J Natl Cancer Inst* **85**: 365–376

Allal A, Kurtz JM, Pipard G, Marti MC, Miralbell R, Popowski Y and Egeli R (1993) Chemoradiotherapy versus radiotherapy alone for anal cancer: a retrospective comparison. *Int J Radiat Oncol Biol Phys* **27**: 59–66

Allal AS, Mermillod B, Roth A, Marti M-C and Kurtz J (1997) Impact of clinical and therapeutic factors on major late complications after radiotherapy with or without concomitant chemotherapy for anal carcinoma. *Int J Radiat Oncol Biol Phys* **39**: 1099–1105

Andersen BL, Anderson B and deProsse C (1989) Controlled prospective longitudinal study of women with cancer: I. Sexual functioning outcomes. *J Consult Clin Psychol* **57**: 683–691

Anderson B and Lutgendorf S (1997) Quality of life in gynecologic cancer survivors. CA Cancer J Clin **47**: 218–225

Anonymous (1996) Epidermoid anal cancer: results from the UKCCCR randomised trial of radiotherapy alone versus radiotherapy, 5-fluorouracil, and mitomycin. UKCCCR Anal Cancer Trial Working Party. UK Co-ordinating Committee on Cancer Research. *Lancet* **348**: 1049–1054

Bartelink H, Roelofsen F, Eschwege F, Rougier P, Bosset JF, Gonzalez DG, Peiffert D, van Glabbeke M and Pierart M (1997) Concomitant radiotherapy and chemotherapy is superior to radiotherapy alone in the treatment of locally advanced anal cancer: results of a phase III randomized trial of the European

© 1999 Cancer Research Campaign

Organization for Research and Treatment of Cancer Radiotherapy and Gastrointestinal Cooperative Groups. *J Clin Oncol* **15**: 2040–2049

Crook J, Esche B and Futter N (1996) Effect of pelvic radiotherapy for prostate cancer on bowel, bladder, and sexual function: the patient's perspective. *Urology* **47**: 387–394

Gelber RD, Goldhirsch A, Cole BF, Wieand HS, Schroeder G and Krook JE (1996) A quality-adjusted time without symptoms or toxicity (Q-TWiST) analysis of adjuvant radiation therapy and chemotherapy for resectable rectal cancer. *J Natl Cancer Inst* **88**: 1039–1045

Kapp KS, Stuecklschweiger GF, Kapp DS, Poschauko J, Pickel H and Hackl A (1997) Carcinoma of the cervix: analysis of complications after primary external beam radiation and Ir-192 HDR brachytherapy. *Radiother Oncol* **42**: 143–153

Klee M, Groenvold M and Machin D (1997) Quality of life of Danish women: population-based norms of the EORTC QLQ-C30. *Qual Life Res* **6**: 27–34

Minsky BD, Cohen AM, Enker WE and Sigurdson E (1992) Phase I/II trial of pre-operative radiation therapy and coloanal anastomosis in distal invasive resectable rectal cancer. *Int J Radiat Oncol Biol Phys* **23**: 387–392

Nigro ND, Vaitkevicius VK and Considine B (1974) Combined therapy for cancer of the anal canal. *Dis Colon Rectum* **27**: 763–766

Papillon J (1974) Radiation therapy in the managenent of epidermoid carcinoma of the anal region. *Dis Colon Rectum* **17**: 181

Papillon J, Monbarbon JF, Gérard JP, Chassard JL and Ardiet JM (1989) Interstitial curietherapy in the conservative treatment of anal and rectal cancers. *Int J Radiat Oncol Biol Phys* **17**: 1161–1169

Perez CA and Brady LW (1992) Overview. In: *Principles and Practice of Radiation Oncology*, 2nd edn, Perez CA and Brady LW (eds), pp. 51–55. Lippincott: Philadelphia

Sedgwick DM, Howard GC and Ferguson A (1994) Pathogenesis of acute radiation injury to the rectum. A prospective study in patients. *Int J Colorectal Dis* **9**: 23–30

Sprangers MA, Taal BG, Aaronson NK and te Velde A (1995) Quality of life in colorectal cancer. Stoma vs. nonstoma patients. *Dis Colon Rectum* **38**: 361–369

Touboul E, Schlienger M, Buffat L, Lefkopoulos D, Pène F, Parc R and Tiret E (1994) Epidermoid carcinoma of the anal canal. Results of curative-intent radiation therapy in a series of 270 patients. *Cancer* **73**: 1570–1579

Williams NS and Johnston D (1983) The quality of life after rectal excision for low rectal cancer. *Br J Surg* **70**: 460–462

Yeoh E, Horowitz M, Russo A, Muecke T, Robb T, Maddox A and Chatterton B (1993) Effect of pelvic irradiation on gastrointestinal function: a prospective longitudinal study. *Am J Med* **95**: 397–406

Yeoh E, Sun WM, Russo A, Ibanez L and Horowitz M (1996) A retrospective study of the effects of pelvic irradiation for gynecological cancer on anorectal function. *Int J Radiat Oncol Biol Phys* **35**: 1003–1010

*© 1999 Cancer Research Campaign*
